# Teacher's Emotional Display Affects Students' Perceptions of Teacher's Competence, Feelings, and Productivity in Online Small-Group Discussions

**DOI:** 10.3389/fpsyg.2021.795708

**Published:** 2022-01-07

**Authors:** Xuejiao Cheng, Han Xie, Jianzhong Hong, Guanghua Bao, Zhiqiang Liu

**Affiliations:** ^1^Key Laboratory of Adolescent Cyberpsychology and Behavior (Ministry of Education), School of Psychology, Central China Normal University, Wuhan, China; ^2^Department of Psychology, School of Humanities and Social Sciences, Hubei University of Medicine, Shiyan, China; ^3^Center of Students' Psychological Development, Department of Students' Affairs, Yancheng Polytechnic College, Yancheng, China

**Keywords:** teacher's emotional display, students' perceptions, students' productivity, online learning, small-group discussion

## Abstract

Teacher's emotions have been shown to be highly important in the quality and effectiveness of teaching and learning. There is a recognized need to examine the essential role of teacher's emotions in students' academic achievement. However, the influence of teacher's displays of emotions on students' outcomes in small-group interaction activities, especially in the online environment, has received little attention in prior research. The aim of the present study was to explore the relationship between teacher's different emotional displays and students' perceptions of the teacher's competence, as well as students' collaborative feelings and productivity in online small-group discussions. Using a three-level between-subjects design, 74 participants were randomly divided into four-member groups comprising a teacher and three other participants. All the groups were asked to discuss an open-ended realistic problem using online software, during which the teacher's display of emotions varied (positive vs. negative vs. neutral). The participants' self-reported questionnaire data (perception of the teacher's competence, students' feeling of pleasure, collaborative satisfaction, and willingness to continue collaborating) and productivity (number of effective ideas expressed within a given time) were measured to compare the participants who were exposed to different emotional displays. As expected, the results showed that the participants who received the teacher's positive emotional display reported that they experienced higher levels of pleasure during the task. However, in contrast to our expectations, those under the negative emotional display condition showed a significantly higher level of productivity in the group task. In addition, compared to emotional display, the participants' perceptions of the teacher's competence were rated significantly higher under the neutral condition, and they reported higher levels of collaborative satisfaction and greater willingness to continue collaborating with their group. The findings have the potential benefit of informing educational practice on whether teachers should display their emotions in a small-group discussion or how they should display emotions following adjustment for the relative aim of the teaching activities.

## Introduction

Collaborative learning can occur in a wide variety of forms, and small-group learning has received researchers' constant attention (Micari and Pazos, 2014; Pai et al., 2015). Multiple students engage in a process together toward a shared learning goal, and, preferably, the group membership ranges from three to five (Wang et al., 2017). A common belief is that meaningful interactions, which refer to the interaction behaviors with personal productivity efforts, can afford opportunities to promote academic outcomes and achieve positive social effects (Tolmie et al., 2010; Micari and Drane, 2011). However, productive behavior among students working does not come easily, which limits the desired effects of small-group learning in educational practice (Slavin, 2014; Wang et al., 2017). To address the gap between the desired value and practice outcomes, various ways to prompt small-group learning have been examined from different perspectives, such as training learners' communication skills, structuring the interaction process, and improving communication (Cohen, 1994; Lou et al., 2001; Jermann and Dillenbourg, 2008; Rummel et al., 2009).

Although numerous studies exist, some researchers consider that inadequate attention is placed on the emotional aspects of small-group learning, which is a pitfall for interaction in such settings, especially in the case where collaborative learning is adjusted from face-to-face education to web-based blended learning through various digital media and collaborative communication tools (Kreijns et al., 2003; Jeong and Hmelo-Silver, 2016; Wang et al., 2017). For example, researchers have proposed that emotional physical cues in online collaboration are relatively lacking in vocal intonation or body language, compared with traditional offline communication (Robinson, 2010). Given this, our study focused on the emotions of teachers and students in the context of communication in online small-group learning, where teacher's emotions are an important factor affecting various aspects of the teaching–learning practice (Meyer and Turner, 2006; Hagenauer et al., 2016; Hansen and Mendzheritskaya, 2017).

### Teacher's Emotional Display and Students' Outcomes

Abundant evidence shows that teacher's emotions greatly influence their well-being (Yin et al., 2016), job satisfaction (Yin, 2015), and professional development (Saunders, 2013). Besides the influences on a teacher's own professional life, researchers have proposed that studies may explore the effect of teacher's emotions as a dynamic factor in interpersonal communication or from the perspective of social interactions (Hagenauer et al., 2016). For example, Meyer and Turner (2006) argue that emotions are ubiquitous in education and are important for understanding instructional interactions. As demonstrated in a series of studies, teacher's emotions, especially positive emotions, are associated with students' reports of their motivation. Other students' outcomes, including academic achievement, well-being, and multidimensional development are all assumed to have relationships with teacher's emotions and interaction behaviors (Rosiek, 2003). Given that students are the direct recipients of the influence of teacher's interaction behaviors and interactions with students are frequently emotionally laden, several aspects have been considered to investigate the role of teacher's emotional display on students (Uitto et al., 2015; Hagenauer et al., 2016).

#### Teacher's Emotional Display and Students' Perceptions of Teacher's Personality

Emotional displays are considered a salient source, and people engaged in social interactions often judge personality traits (e.g., attractiveness, trustworthiness, dominance, and competence) based on certain emotional displays (Keltner and Haidt, 1999; van Kleef, 2009). For example, Hess et al. (2000) used a mix of experimental designs to study the influence of emotional displays on the judgments of 145 Caucasian and Japanese perceptions of dominance and affiliation (Hess et al., 2000). Their results showed that happy emotional displays are tightly linked to a high level of dominance and affiliation and angry emotional displays of people are perceived as low in affiliation and dominance. Such an association between emotional display and perception of personality traits is essential for interpersonal interaction, as it shapes the kinds of strategies to interact with other people, the degree of behavioral productivity in the collaborative process, and whether to continue the interaction (Krumhuber et al., 2007; Van Kleef et al., 2010; Cheng et al., 2013; Fang et al., 2018).

Similarly, studies related to the effects of emotional display on people's perceptions have been conducted in the education environment (Hansen and Mendzheritskaya, 2017; Mendzheritskaya and Hansen, 2019). Researchers have found that the display of emotions, as well as teacher's actions, intentions, or beliefs, have interactional relevance in the context of real education interactions (Frith and Frith, 2006). In other words, students tend to connect their teacher's display of emotions with their teacher's personality traits, which can further affect other learning outcomes related to interaction. For example, several experimental studies examining the effect of teacher's emotional display on students' judgment of the teachers indicate that teacher's “hot” vs. “cold” display correlated with more positive ratings of the teacher, such as “more sociable” and “more humane” (Widmeyer and Loy, 1988; Mendzheritskaya and Hansen, 2019). Other learning-related outcomes, such as student–teacher relationships, teacher's popularity, and teacher's evaluation can be influenced by the connection of emotional display and perceptions of teacher's personality traits (Dong et al., 2021).

However, whether these existing findings can be generalized to small-group learning in an online environment is a question of interest for further investigation. This is because the forms of emotion displayed in online interactions differ from those in traditional communication (Kreijns et al., 2003). In traditional interaction communication, we often use multiple sources of information, such as facial expressions, vocal expressions, and other behaviors to create perceptions about an interaction partner (Scherer, 2003; Oosterhof and Todorov, 2009). However, the sources of emotional information in online interactions, especially emotional information related to physical cues are not well-presented. Therefore, continued investigation in an online environment is necessary.

In addition, many personality traits can be used to judge another person in social interaction (Judd et al., 2005; Todorov et al., 2005). Fiske et al. have proposed the elemental dimensions underlying person perception, namely competence and warmth (Fiske et al., 2002). They suggested that personal perceptions are subject to evolutionary pressures and people must judge other's intentions (relating to warmth) and ability to act on those intentions (relating to competence) (Fang et al., 2018). Given the increasing evidence that the relationship between emotional displays and perceived warmth is robust, more attention should be given to the relationship between emotional displays and perceived competence (Knutson, 1996; Todorov et al., 2005; Fang et al., 2018). Therefore, the first purpose of this present study was to investigate the question of whether or how different displays of teacher's emotions affect students' perceptions of teacher's competence in the interactions of small-group learning in the context of the online environment.

#### Teacher's Emotional Display and Students' Feeling of Pleasure, Satisfaction, and Willingness to Continue Collaborating

The relationship between the emotional display of teachers and students' perceptions has been explored within the framework of emotional transmission (Mendzheritskaya and Hansen, 2019). Emotional transmission (synonym for emotional contagion) means that in daily interactive activities, emotions such as happiness, anger, and sadness can be directly or indirectly “transmitted,” like an infection, from one person to another in a short time (Zeng and Zhu, 2019). Based on emotional transmission theory, it is hypothesized that teacher's and students' feelings or perceptions are interrelated (Becker et al., 2014). These assumptions were further validated by scientific evidence. Some examples are positive emotional displays of teachers (enjoyment or emotional support) that had a good influence on students' feeling of pleasure and reduced students' boredom and frustration (Frenzel et al., 2009).

Nevertheless, empirical support for the relationship between teacher's emotions and students' feelings or perceptions is still scarce (Becker et al., 2014; AlSagri and Ykhlef, 2016). Some researchers contend that little attention has been paid to emotion transmission among group members, which is more complicated (Zheng et al., 2020). For small-group learning in education, when the teacher joins a small group, he or she is indeed a member of the group. The teacher's display of emotions has an influence on the students' feeling of pleasure, and similarly, this process also happens among other group members. Exploring how the teacher's display of emotions is transmitted in small-group interactions in which the teacher is a member of the group can provide empirical and theoretical insight into the emotional transmission and provide further guidance for collaborative practice.

Furthermore, collaborative satisfaction and willingness to continue collaborating are two crucial variables of perceptions toward collaborative small-group learning. Typically, collaborative satisfaction refers to the level at which students' experiences meet their expectations (Alqarni, 2021). The willingness to continue collaborating refers to the degree of the members' willingness to continue collaborating with this group in the future. Previous studies usually used retrospective data from three aspects to assess the group members' perception of collaborative satisfaction: satisfaction with the collaborative atmosphere, satisfaction with the interactive process, and satisfaction with group results (Gladstein, 1984). The total score of the three aspects representing the members' collaborative satisfaction and the responses on a Likert scale was used to assess the group members' willingness to continue collaborating in the usual practice. As pointed out earlier, students tend to connect their teacher's display of emotions with their personality traits or competence while interacting, which consequently impacts their satisfaction with the interaction in the collaboration, as well as whether to continue collaborating (Krumhuber et al., 2007; Van Kleef et al., 2010; Cheng et al., 2013; Fang et al., 2018). In view of this, the second purpose of this study was to reveal the emotion transmission in small-group members during online discussions. Specifically, we aimed to explore how teacher's display of emotions influences students' feelings of pleasure, collaborative satisfaction, and willingness to continue collaborating during emotion transmission.

#### Teacher's Emotional Display and Students' Collaboration Behavior

Recent studies show that emotional displays can influence collaboration among members of a small group according to the contextual meaning of the expressions (de Melo et al., 2021). This conclusion is supported by existing studies. For example, van Doorn et al. conducted three vignette studies focusing on the impact of an interaction partner's emotional display on others' sense-making process of collaborative or completive behavior (Van Doorn et al., 2012). The authors found that compared with happiness or disappointment, anger display will make others experience less collaborative behavior and that people are more willing to express collaborative or prosocial behavior (van Doorn et al., 2015).

However, existing studies on the relationship between emotional display and collaboration mainly employ the SoMi Paradigm, in which collaborative behavior was often represented using the proportion of Player A's (i.e., confederate) selection of “unique object” and “not unique object” (Van Kleef et al., 2010; Van Lange et al., 2013; de Melo and Terada, 2020). It should be noted that the collaborative behavior in this paradigm was conceptualized as a collaborative decision based on the cognition of others' psychological state, emotions, and behavioral intention during interpersonal dynamic processes (Kai et al., 2018). There is some distinction between collaborative decisions and real collaborative behavior, especially in the education environment. Given this, we decided to use the simulation-group discussion task to investigate the effects of emotional display on collaborative behavior (participation behavior during discussions) in an online small group. The level of collaboration was evaluated based on the real-group members' behavioral productivity (the number of effective ideas expressed in the simulation discussion process).

Moreover, when a teacher forms a part of a group in a social context of learning interaction, the display of his/her emotion, either positive or negative, may be regarded as feedback to other group members' actions, thoughts, emotions, needs, attitudes, wills, intentions, etc. (Sarsar, 2017). Many researchers believe that positive teacher's emotional displays are a type of motivational feedback and seem to be conducive to a range of desirable outcomes, including encouraging students to seek to learn more about a specific topic or helping students to remain engaged in the small-group learning process (Kim and Keller, 2007; Sarsar, 2017; de Melo and Terada, 2020). However, there are some exceptions. Results from the study of the negative emotional display indicate that some negative emotions, such as anger display, can have positive effects on students, as it signals high expectations for the students (Butler, 1994; Frenzel et al., 2020). Considering that motivation and teacher's expectations are both important factors affecting students' behavior, the inconsistent conclusions warrant continued empirical attention to the relationship between teacher's emotional display and student outcomes, especially in a collaborative environment. Therefore, the third purpose of this study was to explore the open question of how different emotional displays affect behavioral productivity among group members in small-group learning.

In summary, productive collaborative learning is rarely spontaneous, and the teacher's display of emotions is a powerful educational tool that may enhance various aspects of students in small-group work. However, there is limited research on teacher's display of emotions and its impact on students' outcomes in online education, especially in text-based synchronous online small-group discussions. This study contributes to closing this gap in the literature. Specifically, the following issues were addressed in this study:

1. How do different emotional displays of teachers affect students' perceptions of teacher's competence in interactions during online small-group learning?
2. How do teacher's displays of emotions influence students' feelings of pleasure, satisfaction, and willingness to continue collaborating in a small-group discussion when the teacher is a member of the group?
3. How do teacher's different emotional displays affect students' productivity among group members in small-group discussions?

### The Present Study

The present study tested the influence of teacher's display of emotions on students' judgments regarding the teacher's competence, collaborative perceptions (feeling of pleasure during the group task, collaborative satisfaction, willingness to continue collaborating), and productivity in online group interaction. We manipulated the teacher's display of emotions (positive vs. negative vs. neutral) using emoticons from a software platform and recorded behavioral productivity during a creative discussion task (an open-ended realistic problem) in an online small group. Previous studies on the relationship between teacher's display of emotion and students' perceptions in a face-to-face environment were used as a reference (Van Kleef et al., 2010; de Melo and Terada, 2020). Given that the cognitive process of collaboration in online groups is similar to that of face-to-face groups, based on the theories and the results of previous studies discussed, the following hypotheses were developed: H1: Compared with an emotional display, participants experiencing neutral emotional display may rate the levels of teacher's competence higher. H2: Compared with the neutral emotional display condition and the negative condition, participants who experience the teacher's positive emotional display may report higher levels of pleasure, collaborative satisfaction, and stronger willingness to continue collaborating. H3: Compared with neutral emotional display, participants who experience a teacher's emotional display (positive or negative) may present a higher level of behavioral productivity in the group task.

## Methods and Materials

### Participants and Design

Ninety Chinese university students were recruited through voluntary advertising (19 men, age: 21.28 ± 3.51 years). The teacher's emotional display was manipulated, and the participants were randomly assigned to either the positive emotional display condition (*n* = 30), the negative emotional display condition (*n* = 30), or the control condition (*n* = 30). We defined the sample size based on an earlier study that examined the emotional display effect on person perception (Saito et al., 2019). The volunteers were studying a wide range of disciplines in four universities in different cities. Regrettably, 16 participants dropped out of the experiment separately due to the problems of timing, connectivity, or other personal reasons. After deleting missing and invalid data, we obtained usable data from 74 participants: positive display condition (*n* = 24), negative display condition (*n* = 24), and control condition (*n* = 26). Informed consent was obtained from each participant prior to the experiment. Each participant was paid 10 – Y for participation. The study protocol was approved by the local Academic Committee.

A between-subjects design was used in this study. The participants were randomly assigned to groups of four according to the existing group size research, referring to small-group problem solving (Gu et al., 2020). Although the participants were told that they would perform the task in fours with a teacher, they participated in the experiment collaborating with an experimental assistant who masqueraded as a teacher and all the information of the pretend teacher presented in the group was preprogrammed. The pretend teacher was a 27-year-old male student majoring in psychology, who was responsible for displaying different emotions during the group task. This was done to ensure that the quantity and quality of the information presented by the pretend teachers were comparable for each participant.

### Materials

#### Manipulation of Emotional Display

Common emojis in interactive digital communication were used for the manipulation of teacher's display of emotions. This was done because there was an assumption that using emojis is a possible way to convey emotions in text-based online communication and compensate for the lack of nonverbal communicative cues (Chatzichristos et al., 2020). To select the appropriate emojis to be used as different emotional display materials, we drew on one of the first and most well-known emoji sentiment lexicons that were created based on the context of 1.6 million tweets (Kralj Novak et al., 2015). More importantly, this type of emoji sentiment lexicon is commonly applied in China's daily communication software. According to the emojis sentiment valance and familiarity, three positive and three negative face emojis were preselected by two research assistants first. Then, 30 volunteers recruited randomly through an advertisement from the university were asked to evaluate the six emojis according to their emotional content using a questionnaire consisting of 12 items rated on a five-point scale. Examples of the items in the questionnaire were: “If a teacher sends an emoji like this, what emotion do you think the teacher is expressing? and “If you receive an emoji like this from a teacher, what emotion will you experience?” (1, very negative; 5, very positive). The first question was set to avoid misunderstanding of the emotional valance of the teacher among the participants, and the second question aimed to ensure that the participants' emotions were aroused by the emotional information.

Table 1 shows the emojis preselected by two research assistants and the mean score and SD reported by 30 Chinese students. The first two columns in Table 1 show the emojis used and their descriptions. The third column includes the emotion scores of the teachers evaluated by the students when they used an emoji (Q1), while the last column shows the scores of students' emotions aroused by the emoji representing the teacher's emotional display (Q2). According to the data in Table 1, the “smiling face with a handclap” and the “sad face with the corners of the mouth turned downward” were chosen as the final experimental materials.

**Table 1:** Emotion scores of the different emojis used in this study.

**Emoji**	**Description**	**Q1**		**Q2**	
		***M***	***SD***	***M***	***SD***
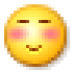	Smiling face with corners of mouth turned up	4.50	0.100	4.58	0.099
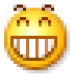	Smiling face with open mouth	4.73	0.089	4.65	0.095
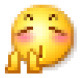	Smiling face with handclap	**4.81**	0.096	**4.69**	0.108
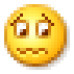	Sad face with frown	1.81	0.096	1.96	0.087
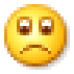	Sad face with corners of mouth turned down	**1.35**	0.110	**1.42**	0.126
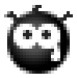	Sad face with tear	1.69	0.133	1.96	0.152

*Q1, Teacher's emotion evaluated by the students; Q2, Student's emotion aroused by the emoji. The values of emojis were chosen for the final experimental materials are bolded*.

For the positive emotional display, the pretend teacher presented information in the group such as “Please continue expressing your ideas + a positive emoji (smiling face with a handclap).” For the negative emotional display, the pretend teacher presented information in the group such as “Please continue expressing your ideas + a negative emoji (sad face with corners of the mouth turned downward).” For the control condition, the pretend teacher only presented information in the group such as “Please continue expressing your ideas” without any emoji, and this was assessed as the sentence best representing the neutral emotion displayed by the 30 Chinese students.

The emotional display information was provided during the group discussions process 2, 4, 6, and 8 min after the experiment, which was constant among all groups. This was done to ensure that the teacher was involved in the entire process of the group task. Moreover, we decided to present the emotional display information at fixed times after the beginning of the experiment for several reasons. First, it would be difficult for the teacher to determine the moment to display the emotion. Second, it would be difficult for the teacher to evaluate the quality of ideas. Third, to exclude the potential effect of the frequency of emotional display, the emotion was displayed only four times.

#### Experimental Task

The study was conducted online through a software platform (QQ), which is widely used in educational settings and daily communication in China (Zhang et al., 2021). The users can send messages to other people in a QQ group, and there is an area used to display group membership information or messages posted by their group members. The participants were invited to participate in an online small-group study conducted by the research team. If the participants agreed to enroll in the online group study, a research assistant invited them to join the preset online chat room (QQ group), directing them to the experimental environment.

The instructions for the group task and rules were clarified in the online small group. Specifically, the group task was to discuss the following topic for 10 min: “How to improve college students' dormitory life satisfaction.” This is a typical sample of the Realistic Presented Problems (RPP), which was of potential interest to the participants. Each group was required to generate as many novel ideas about the topic as possible, and all the ideas presented in the group were recorded for the next steps in the analysis. In the discussion process, the generation of ideas and feedback to each other often occur simultaneously. Hence, it is reasonable to speculate that the participants might also display emotions toward the feedback, especially if they pretend that the teachers display emotional information that looks like feedback during the group discussion. To avoid this, the participants were encouraged to improve on and combine the ideas generated by their group members but not to evaluate each other's ideas. Based on our observation, the participants did not evaluate each other's ideas. Therefore, in this study, the potential contaminant effect of the display of emotions from the participants was excluded.

#### Measurements

##### Basic Information Questionnaire

The basic information questionnaire was divided into two parts. The first part of the questionnaire captured the participants' demographic characteristics including gender, age, and major. The second part was aimed to measure the participants' positive and negative emotions within 2 weeks as the emotional baseline. A Chinese modified version of the Positive and Negative Affect Schedule (PANAS) developed by Watson et al. (1988) was adopted. The scale consists of eight items describing various emotions in terms of four positive (happy, excited, enthusiastic, and inspired) and four negatives (upset, irritable, afraid, and scared) affective descriptors (Dou et al., 2018). In this study, each question was scored on a seven-point Likert-type scale, and Cronbach's alpha reliabilities were all acceptable, 0.73 for positive items and 0.69 for negative items.

##### Manipulation Check Questionnaire

The participants were asked to respond to two manipulation check questions on a five-point Likert scale regarding the emotional display of the pretend teacher. The valence-arousal space is a typical two-dimensional scale used to characterize emotions (Russell, 1980). The first question asked the participants to estimate the emotional valence of the teacher's emotional display they experienced. The second question asked the participants to estimate the emotional arousal of the teacher's emotional display they experienced by responding to the item, “To what extent were you experiencing the emotion of the teacher during the group task.”

##### Questionnaire to Assess Participants' Perception of the Teacher

We used the items derived from prior research related to perceptions of competence to measure the participants' perception of the teacher who presented emotional information in the experiment (Fiske et al., 2002). The use of this measurement has previously been reported by Abele et al. (Abele and Wojciszke, 2014). There were 12 key trait words in the items of the scale, of which six were typical competence trait words (e.g., confident, intelligent, and competent). The participants indicated their degree of agreement or disagreement with each item on a five-point Likert scale ranging from 1 (strongly agree) to 5 (strongly disagree). The reliability coefficient (Cronbach's alpha) for the subscale used in this study was 0.95, which indicated satisfactory reliability.

##### Questionnaire Related to Participants' Feelings in the Collaboration

In this study, the participants' perceptions influenced by different teacher's emotional displays consisted of three parts: the participants' feeling of pleasure, collaborative satisfaction, and willingness to continue collaborating. First, to assess the effect of the teacher's display of emotions on participants' feelings in the collaboration, the participants were asked to recall the group process at the end of the experiment and rate the feeling of pleasure/displeasure they had experienced during the group task (Jones and Ekkekakis, 2019). The feeling of pleasure (displeasure–pleasure) was assessed using the modified Feeling Scale (FS) (Hardy and Rejeski, 1989). The FS is a single-item, five-point Likert-type scale, where 1 denotes “very bad” and 5 denotes “very good.” Higher scores represent more positive emotion. Second, the three “collaborative satisfaction” items (slightly modified from previous studies) on a seven-point Likert-type scale were used to measure the participants' collaborative satisfaction (e.g., “I am very satisfied with the collaborative atmosphere/interactive process/group results”) (Gladstein, 1984). Cronbach's alpha reliability was 0.95, and the mean score of the three items represented the degree of collaborative satisfaction. Finally, the willingness to continue collaborating was measured using a single item (with the question: To what extent are you willing to continue collaborating with this group in the future?). Response options for this item ranged from very unwilling to very willing on a seven-point Likert scale.

##### Productivity Measure

The evaluations proposed in online learning environments have been diverse in previous studies. Some studies have used basic behavioral data to reflect the participants' outcomes of online collaboration, such as time spent, the number of visits, or the ideas given by students (Palmer et al., 2008; Hamann et al., 2009). In this study, the number of effective ideas expressed in the group task within a given time by each participant was used as an indicator of the behavioral productivity of the group members. Effective ideas were expressed as ideas directly linked to problem-solving, that is, problem-irrelevant information (i.e., I am not satisfied with the dormitory in our school!) and duplicated ideas (two consecutive similar thoughts in a group) were eliminated. Two coders were trained to assess the number of effective ideas, and disagreements were resolved through discussion.

### Procedure

**Figure 1:**
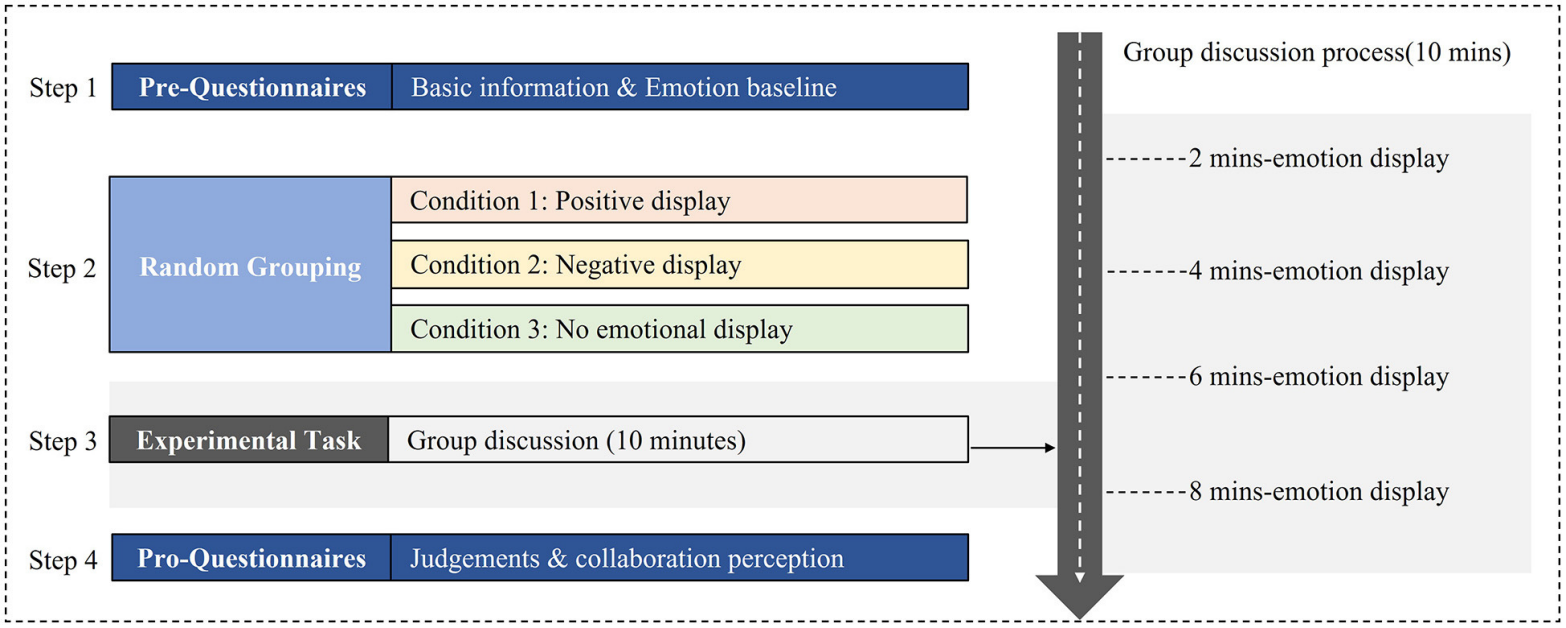
Diagram of experiment design and procedure.

Upon arriving on the QQ software platform, participants were told that they would be grouped with other participants and a teacher who had already been waiting for them online in the QQ group. Then, the experimenter introduced the group task to the group members as previously introduced. To appear real, everyone in the QQ group was asked to make a brief self-introduction. After the instruction section, the group worked on the task for 10 min, while the pretend teacher displayed different emotions during the process. This procedure was repeated under three different emotional display conditions. When the online small-group task was completed, a short self-report questionnaire was presented to each participant to measure the perception of the teacher and their collaboration perceptions. A diagram of the experimental design and procedure is shown in Figure 1.

## Results

To compare the effect of teacher's emotional display on participants' outcomes in online small-group discussions, we conducted an ANOVA to test differences in judgments, perceptions, and behavioral productivity across the experimental groups under the three conditions. Descriptive statistics (means, *M*, and *SD*) of the participants in the experiment are shown in Table 2. Furthermore, linear regressions were conducted to examine the relationship between the judgments toward the teacher, students' collaborative perceptions, and behavioral productivity to further understand how the differences in the teacher's display of emotions influenced group members in the online small-group discussions. The correlation results for each variable are presented in Table 3.

**Table 2:** Results of descriptive statistics and univariate ANOVAs on variables.

**Dependent**	**Conditions**	***N***	***M***	***SD***	***F***	***η^2^***
Perceptions of teacher's competence	Positive	24	3.48	0.86	7.63^*^	0.18
	Negative	24	2.80	0.95		
	Neutral	26	3.76	0.94		
Feeling of pleasure	Positive	24	4.04	0.81	11.66^**^	0.26
	Negative	24	3.04	0.69		
	Neutral	26	3.96	0.99		
Collaborative satisfaction	Positive	24	5.35	1.01	10.28^**^	0.23
	Negative	24	4.28	1.30		
	Neutral	26	5.71	0.99		
Willingness to continue collaborating	Positive	24	5.63	0.92	4.10^*^	0.11
	Negative	24	5.00	1.53		
	Neutral	26	5.92	1.10		
Productivity in collaborative task	Positive	24	6.04	2.66	3.35^*^	0.09
	Negative	24	7.79	3.35		
	Neutral	26	5.65	3.19		

** *p < 0.01*,

* *p < 0.05*.

**Table 3:** Correlation results of dependent variables in the experiment.

	**Competence**	**Feeling of pleasure**	**Collaborative satisfaction**	**Willingness to continue collaborating**	**Behavioral productivity**
Competence					
Feeling of pleasure	0.681^**^				
Collaborative satisfaction	0.553^**^	0.572^**^			
Willingness to continue collaborating	0.471^**^	0.675^**^	0.432^**^		
Productivity in collaborative task	−0.153	−0.128	−0.109	−0.073	

** *p < 0.01*.

### Manipulation Check

We analyzed the manipulation of teacher's emotional displays. Univariate ANOVA using different emotional display conditions as the between-subjects factor was performed on participants' perceived teacher's emotional valence and arousal. The results showed a significant main effect of teacher's emotional displays on the teacher's perceived emotional valence [*F*_(2,71)_ = 34.62, *p* < 0.001, η^2^ = 0.49], and no difference in teachers perceived emotional arousal [*F*_(2,71)_ = 0.60, *p* > 0.05, η^2^ = 0.02]. The result confirmed that the manipulation was successful.

### Participants' Perception of Teacher's Competence

A one-way ANOVA using different emotional display conditions as the between-subjects factor was performed on the participants' perception of the teacher's competence, and gender and age were added to the ANOVA model as covariates. The results showed that there was a significant main effect of different emotional displays on participants' perception of competence toward the teacher [*F*_(2,71)_ = 7.63, *p* < 0.05, η^2^ = 0.18]. The *post-hoc* test showed that the participants' perception of the teacher's competence under the control condition (*M* = 3.76, *SD* = 0.94) was significantly higher than that under the negative emotional display condition (*M* = 2.80, *SD* = 0.95; *p* < 0.01), and there was no significant difference between the control and positive conditions (*M* = 3.48, *SD* = 0.86; *p* > 0.05). Thus, Hypothesis 1 was supported.

### Participants' Feeling of Pleasure, Satisfaction, and Willingness to Continue Collaborating

An ANOVA with different emotional displays as the between-subjects factor was performed on students' feeling of pleasure in the collaboration, and emotional baselines of participants within 2 weeks as well as gender and age were added to the ANOVA model as covariates. As expected, the results showed a significant main effect of different emotional displays on the students' feeling of pleasure [*F*_(2,71)_ = 11.66, *p* < 0.001, η^2^ = 0.26]. The *post-hoc* test showed that the students' feeling of pleasure under the positive emotional display condition (*M* = 4.04, *SD* = 0.81) was significantly higher than under the negative emotional display condition (*M* = 3.04, *SD* = 0.69; *p* < 0.001). There was no significant difference between the positive and control conditions (*M* = 3.96, *SD* = 0.99; *p* > 0.05). Thus, Hypothesis 2 was partially supported.

In addition, the participants' perceptions of collaborative satisfaction were analyzed using a one-way (between-subjects factor: different emotional display) ANOVA. There was a significant main effect of different emotional displays on the participants' perceptions of collaborative satisfaction [*F*_(2,71)_ = 10.28, *p* < 0.001, η^2^ = 0.23]. The *post-hoc* test showed that the perceptions of collaborative satisfaction under the positive emotional display condition (*M* = 5.35, *SD* = 1.01) was significantly higher than under the negative emotional display condition (*M* = 4.28, *SD* = 1.30; *p* < 0.001). There was no significant difference between the positive and control conditions (*M* = 5.71, *SD* = 0.99; *p* > 0.05). To further explore the relationship between collaborative satisfaction and other variables, a linear regression analysis was conducted. Only competence (β = 0.38, *B* = 0.48, *p* = 0.029 < 0.05, *R*^2^ = 0.529) was significant. The results indicated that better perception of teacher's competence predicted higher perceptions of collaborative satisfaction with online small-group discussions.

Moreover, a one-way ANOVA using different emotional displays as the between-subjects factor was performed on the willingness to continue collaborating, and gender and age were added to the ANOVA model as covariates. The results showed a significant main effect of emotional display on willingness to continue collaborating [*F*_(2,71)_ = 4.10, *p* < 0.05, η^2^ = 0.11]. The *post-hoc* test showed that the willingness to continue collaborating under the negative emotional display condition (*M* = 5.00, *SD* = 1.53) was significantly lower than under the control condition (*M* = 5.92, *SD* = 1.10; *p* < 0.05). There was no significant difference between the control and positive conditions (*M* = 5.63, *SD* = 0.92; *p* > 0.05). To further explore the relationship between the willingness to continue collaborating and other variables, a linear regression analysis was conducted. Only the perceptions of collaborative satisfaction (β = 0.62, *B* = 0.63, *p* = 0.000 < 0.01, *R*^2^ = 0.478) were significant. The results indicated that students with higher satisfaction had a stronger willingness to continue collaborating with their group members.

### Participants' Productivity in the Small-Group Discussion

A one-way ANOVA using the different emotional displays as the between-subjects factor was performed on the behavioral productivity of participants in the group task. The results showed a significant main effect of emotional display on the participants' collaborative behavioral productivity [*F*_(2,71)_ = 3.35, *p* < 0.05, η^2^ = 0.09]. The *post- hoc* test showed that the behavioral productivity of the participants under the negative emotional display condition (*M* = 7.79, *SD* = 3.35) was significantly higher than under the control condition (*M* = 5.65, *SD* = 3.19; *p* < 0.05). However, there was no significant difference between the control and positive conditions (*M* = 6.04, *SD* = 2.66; *p* > 0.05). The result was contrary to Hypothesis 3 and indicated that those under the negative emotional display condition were more likely to present a higher level of behavioral productivity in the group task.

## Discussions and Limitations

It is well-known that teacher's display of emotions influences various teaching–learning outcomes. However, there is limited research on the role of teacher's emotional display in small-group learning from the perspective of interaction, especially in text-based synchronous online learning environments. This study helps to close this gap in the literature. Thus, practitioners, researchers, and teachers will be provided with useful insights into how teacher's emotional displays influence students' perceptions and behavior productivity. In this study, we provided real-time adaptive emotional display as feedback to emotionally support interaction during small-group discussions. This study aimed to investigate how different teacher's emotional displays affect students' perceptions of the teacher, students' collaboration, and productivity in an online group. The participants were asked to discuss one realistic problem in four-member online groups, with a teacher as a member of each group.

### Teacher's Display of Emotions and Students' Perception of the Teacher's Competence

The results regarding the participants' judgment of the teacher's competence revealed that students gave significantly higher scores on the teacher's competence under the neutral emotional display condition. This result confirms the conclusion that the emotion expressed by a teacher indeed affects the students' perception of the teacher's personality (Mendzheritskaya and Hansen, 2019). However, there exist some differences from previous studies on the relationship between specific emotional displays and students' perceptions. According to previous studies, a teacher's negative emotional displays mostly result in a more negative perception of the teacher's personality, and a teacher's positive emotional display is always connected with a more positive perception of the teacher's personality (e.g., more conscientious, more cautious, or more understanding). However, our results showed otherwise. Under the “control condition,” when a neutral emotion was displayed by the teacher, students in the online group rated the teacher as being more competent in comparison to their counterparts under emotional display conditions, whether positive display or negative display.

Culture could be the reason for the result that teachers displaying neutral emotion in the group were rated higher in terms of competence (Hansen and Mendzheritskaya, 2017; Mendzheritskaya and Hansen, 2019). According to Markus and Kitayama (1991) theory of culture and self, people from independent cultures (e.g., American culture) tend to value free and open emotional displays. In contrast, those from Eastern cultures (e.g., Chinese culture) tend to value emotional self-control, emotional restraint, and emotional suppression in pursuit of interpersonal harmony (Markus and Kitayama, 1991). For example, a qualitative study of Chinese people found that they suppressed both negative and positive emotions for reasons such as controlling impulse rationally to prevent hurting others, allowing time to process what was going on, or avoiding showing off too much (Chiang, 2012). From the Chinese cultural perspective, emotional suppression may be appropriate for group harmony, and those who display neutral emotion in group activities may be regarded as having a high ability (Wei et al., 2013). Overall, the judgment of personality traits is influenced by culture-specific patterns and personal values within nations (Schmitt et al., 2007; Heine and Buchtel, 2009). The neutral emotional display of the teacher in teaching-learning activities may be more in line with students' expectations of an ideal teacher or a professional teacher in this study.

### Teacher's Emotional Display and Students' Feeling of Pleasure, Satisfaction, and Willingness to Continue Collaborating

The results revealed that the students' feeling of pleasure under the positive emotional display condition was significantly higher than under the negative emotional display condition. The results extend the conclusions of previous studies, suggesting that emotional transmission occurs not only in face-to-face traditional communication (Frenzel et al., 2009) but also in online collaborative groups in the context of education. In addition, our results showed that the participants reported higher levels of collaborative satisfaction and stronger willingness to continue collaborating with their group under the neutral emotional display condition than under the other two emotional display conditions. The most likely reason for this result is the influence of students' perception of teacher's personality traits on students' interpersonal interactions. As mentioned above, the teacher's display of emotion influences students' perception of teachers, which is essential for interpersonal interaction in small-group learning. For example, when a person is judged to be dominant and aggressive, he or she is less likely to be chosen as a group member to work on a collaborative project; conversely, when a person is judged to be competent or trustworthy, we are more inclined to seek help from them when we are in trouble or collaborate with them (Krumhuber et al., 2007; Van Kleef et al., 2010; Cheng et al., 2013; Fang et al., 2018). The result of linear regression from this study that better perception of teacher's competence predicted higher perceptions of collaborative satisfaction confirms our reasoning. In short, the results regarding the influence of teacher's display of emotions on students' collaboration perception indicated that students tend to connect their teacher's emotions with their personality, which may consequently impact students' collaborative satisfaction and willingness to continue collaborating in teaching and learning activities (Mendzheritskaya and Hansen, 2019).

### Teacher's Display of Emotions and Students' Productivity in the Small-Group Discussion

Moreover, we explored the effect of teacher's display of emotions on students' behavioral productivity in online small-group learning. The results showed that those under the negative emotional display condition showed significantly higher levels of behavioral productivity during the group task followed by students under the positive emotional display condition, and finally, those under the neutral emotional display condition. Our findings are consistent with previous observations that emotional display or emotional reactions are powerful tools to keep students more motivated, remain engaged in the learning process, and facilitate better collaboration in education (Meyer and Turner, 2006; Maier et al., 2016; Sarsar, 2017). In other words, the teacher's emotional display improved students' levels of motivation, resulting in a high level of behavior productivity. However, it is noteworthy that the level of behavior productivity of students under the negative emotional display condition was significantly higher than that under the positive condition. A possible reason for this result could be that teacher's negative emotional display may result in students' unpleasant feelings, such as anxiety or stress, which is a complex emotional state. Appropriate anxiety is conducive to enhancing the response speed and alertness of the brain, thus improving the task behavioral productivity (Hordacre et al., 2016; Chen and Beck, 2019). In addition, this result that students under the negative emotional display condition generate the most ideas can also be interpreted in the context of the dual-process model of creativity proposed by De Dreu et al. (2008). The model suggests that positive emotional states enhance creativity through flexibility, while negative emotional states can enhance creativity because they stimulate emotional persistence. Namely, the teacher's negative emotional display connects with students' negative emotions, and this connection causes them to engage in more creative activities (generate more ideas and show a higher level of behavior productivity) by improving cognitive persistence.

Overall, the principal finding of this study is that teacher's emotional display influences students' perception of teachers, collaboration perceptions, and behavior productivity in online small-group learning, and researchers and teachers would provide useful insights regarding how to use them. These findings have practical implications. If the goal of the discussion activity is to bring students the experience of pleasure, then the teacher involved in the online small-group learning needs to present his/her positive emotions. If the activity's goal is to improve student participation in the discussion, then only the teacher's negative emotion is shown. Note that, if the teacher's aim is to establish well-functioning relationships among the group members, then the neutral emotional display is better for the teacher's competence evaluation, collaborative satisfaction, and willingness to continue collaborating in the Chinese cultural context.

## Limitations

The present study has several limitations. First, the participants in the online group were unfamiliar with their group members prior to the experiment, which might not coincide with reality. In a real educational setting, students in a small-learning group are supposed to be acquainted with their teacher and group members. Therefore, the possible effects of familiarity on the relationship between teacher's emotional displays and students' perceptions and behavioral productivity should be further tested. Second, although the sample size was similar to other studies that examined the emotional display effect on person perception (Saito et al., 2019), a larger sample would help to better understand the effect of teacher's emotional display on the students' outcomes at the group level and better understand the possible interference of other variables (such as individual differences), thus achieving larger effect sizes. In addition, previous studies have shown that gender composition can influence group collaborative behavior (Liu et al., 2017). In this study, the number of male participants was very small. More male participants will need to be recruited to constitute more groups in terms of different gender compositions. In addition, the participants in this study were recruited from a wide range of disciplines; in other words, the students' different academic backgrounds and experiences might also have an impact on synthesis findings. Thus, some meaningful extensions can be made in the future to focus more on the discipline's students' characteristics and their impact on the outcomes related to the teacher's emotional display. Finally, presenting the emotional display information at fixed times irrespective of the actual ideas being raised at that time might lead to confusion (e.g., a positive emotional display to poor ideas and negative emotional display to good ideas). While the rationale for doing so was given in the text as mentioned before, a more appropriate way to present emotional display information should be adopted in future studies so that the potential effect of feelings of confusion could be ruled out.

## Conclusions

By exploring how the display of teacher's emotions affects students' outcomes in online small-group discussions, the current study provides crucial findings for future studies and an understanding of the relationship between different teacher's emotional display and students' perception of the teacher's competence, students' collaborative perceptions, and behavior productivity in online small-group discussions. Using an empirical method, this study found that students who received a positive emotional display experienced a higher level of pleasure during the task. Notably, students who received the teacher's negative emotions showed a significantly higher level of behavioral productivity in the group task. In addition, the levels of students' judgment of the teacher's competence, as well as collaborative satisfaction and willingness to continue collaborating, were higher when they received neutral emotional display. Thus, the study offers some practical implications on whether teachers should display their emotions in a small-group discussion or how they display emotions according to the aim of the teaching activities.

## Data Availability Statement

All data included in this study are available upon request by contact with the first author XC (cxuejiaohappy@foxmail.com).

## Ethics Statement

The studies involving human participants were reviewed and approved by Central China Normal University. The patients/participants provided their written informed consent to participate in this study.

## Author Contributions

XC, HX, and JH contributed to the conception and design of the study. GB and ZL coordinated the data collection. HX performed the statistical analysis and XC wrote the first draft of the manuscript. All the authors contributed to manuscript revision and approved the submitted version.

## Funding

This study was supported by the Research Funds of Philosophy and Social Science Major Research Project in Jiangsu Province (2020SJZDA166), the National Natural Science Foundation of China (61877025) and Research Funds of Hubei Provincial Department of Education (18Z404).

## Conflict of Interest

The authors declare that the research was conducted in the absence of any commercial or financial relationships that could be construed as a potential conflict of interest.

## Publisher's Note

All claims expressed in this article are solely those of the authors and do not necessarily represent those of their affiliated organizations, or those of the publisher, the editors and the reviewers. Any product that may be evaluated in this article, or claim that may be made by its manufacturer, is not guaranteed or endorsed by the publisher.
